# Supplementary material for: The covariance perceptron: A new paradigm for classification and processing of time series in recurrent neuronal networks
Source: PLoS Comput Biol. 2020 Oct 12;16(10):e1008127. doi: 10.1371/journal.pcbi.1008127 (PMC7595646; doi:10.1371/journal.pcbi.1008127)
Supplement: S1 Appendix — Simulations based on the analytical input-output mapping for network with trained afferent and recurrent connectivities and spatio-temporal covariances. (PDF) [file pcbi.1008127.s001.pdf]

Supplementary Results.

The covariance perceptron: A new paradigm for classification and processing of time series in recurrent neuronal networks

Matthieu Gilson[1,2◑], David Dahmen[2◑], Rubén Moreno-Bote[1,3], Andrea Insabato[4], Moritz Helias[2,5]

1 Center for Brain and Cognition, Universitat Pompeu Fabra, Barcelona, Spain
2 Institute of Neuroscience and Medicine (INM-6) and Institute for Advanced Simulation (IAS-6) and JARA Institute Brain Structure-Function Relationships (INM-10), Jülich Research Centre, Jülich, Germany
3 ICREA, Barcelona, Spain
4 IDIBAPS, Universitat de Barcelona, Barcelona, Spain
5 Department of Physics, Faculty 1, RWTH Aachen University, Aachen, Germany

◑These authors contributed equally to this work.
* matthieu.gilson@upf.edu

Shaping output spatio-temporal covariances

As shown in Fig. I-A, we want to tune both B and A to obtain a desired spatio-temporal structure in output. We consider inputs x_k^t with spatial covariances only (since $P^1 = 0$) to be mapped to spatio-temporal covariances for y_i^t. For this purpose, we generalize Eq. (11) to calculate the weight updates for A and B from the errors of both Q^0 and Q^1:

$$\begin{aligned} \Delta B_{ik} &= \eta_B \left[(\bar{Q}^0 - Q^0) \odot \frac{\partial Q^0}{\partial B_{ik}} + (\bar{Q}^1 - Q^1) \odot \frac{\partial Q^1}{\partial B_{ik}} \right] , \\ \Delta A_{ij} &= \eta_A \left[(\bar{Q}^0 - Q^0) \odot \frac{\partial Q^0}{\partial A_{ij}} + (\bar{Q}^1 - Q^1) \odot \frac{\partial Q^1}{\partial A_{ij}} \right] . \end{aligned} \quad \text{(S1)}$$

The matrix derivatives are given by Eqs. (30), (32), (33) and (34) in Methods (main text) while setting $P^1 = P^{-1\mathrm{T}} = 0$, which read in matrix form:

$$\begin{aligned} \frac{\partial Q^0}{\partial B_{ik}} &= A \frac{\partial Q^0}{\partial B_{ik}} A^{\mathrm{T}} + U^{ik} P^0 B^{\mathrm{T}} + B P^0 U^{ik\mathrm{T}} , \\ \frac{\partial Q^1}{\partial B_{ik}} &= A \frac{\partial Q^1}{\partial B_{ik}} A^{\mathrm{T}} + A U^{ik} P^0 B^{\mathrm{T}} + A B P^0 U^{ik\mathrm{T}} , \\ \frac{\partial Q^0}{\partial A_{ij}} &= A \frac{\partial Q^0}{\partial A_{ij}} A^{\mathrm{T}} + V^{ij} Q^0 A^{\mathrm{T}} + A Q^0 V^{ij\mathrm{T}} , \\ \frac{\partial Q^1}{\partial A_{ij}} &= A \frac{\partial Q^1}{\partial A_{ij}} A^{\mathrm{T}} + V^{ij} Q^1 A^{\mathrm{T}} + A Q^1 V^{ij\mathrm{T}} + V^{ij} B P^0 B^{\mathrm{T}} . \end{aligned} \quad \text{(S2)}$$

The key to evaluate the weight update for A is seeing that the third and fourth lines correspond to the discrete Lyapunov equation that can be solved at each optimization step. As before, we randomly draw

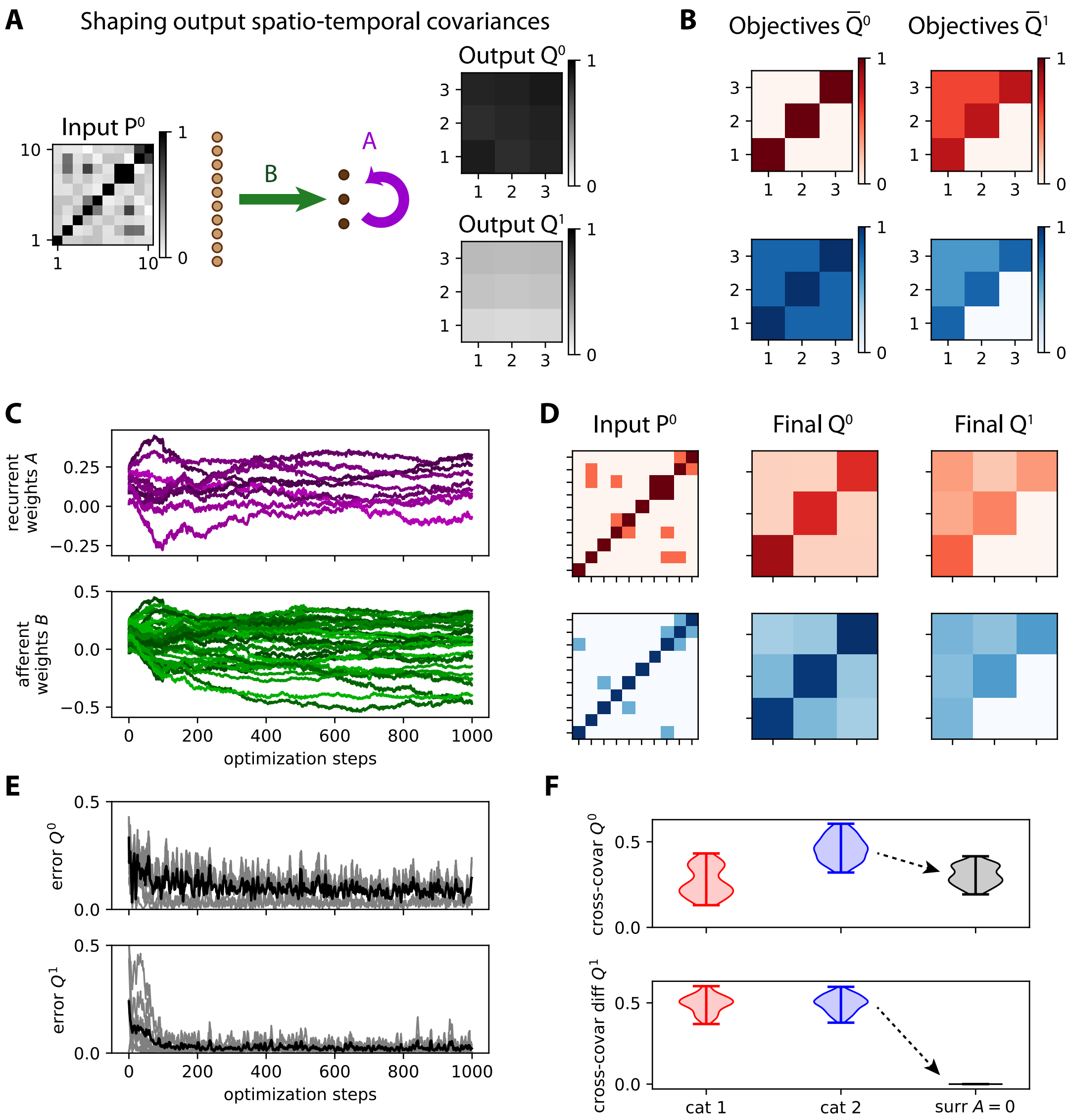

Fig I. Shaping output spatio-temporal covariances with both afferent and recurrent connectivities. **A:** Network architecture with $m = 10$ input nodes and $n = 3$ output nodes, the latter being connected together by the recurrent weights A (purple arrow). **B:** Objective covariance matrices for two categories (red and blue). The categories differ by their respective $\bar{Q}^0$, but they have the same $\bar{Q}^1$. **C:** Evolution of the afferent and recurrent weights (green and purple traces, respectively). **D:** Two examples after training of output patterns Q^0 and Q^1 in response to two input patterns P^0, among the 5 in each category. **E:** Evolution of the error for the two output covariance matrices. **F:** After training, the covariances in Q^0 allow for the discrimination between the two categories, while the structure of Q^1 is similar for the two categories (as imposed by the objectives). The plot is similar to Fig. 4 (main text). The black surrogate corresponds to forcing $A = 0$ with the trained B and presenting the blue inputs, demonstrating that the trained A is important in shaping the output structure.

10 input patterns to be classified into 2 categories of 5 each, whose objective matrices Q^0 and Q^1 are represented in Fig. I-B. A positive outcome is that the weight updates lead to rather stable learning dynamics, even for the recurrent connectivity in Fig. I-C. The stability of ongoing learning while leaving classification aside is examined in Fig. 7 in the main text. Meanwhile, the errors for both Q^0 and Q^1 decrease and eventually stabilize close to zero in Fig. I-E.

After training, the network maps the input patterns P^0 in the desired manner for Q^0 and Q^1, see the two examples in Fig. I-D and the robustness test in Fig. I-F —in a similar manner to Fig. 4 (main text). The surrogates (black distribution in Fig. I-F) correspond to setting $A = 0$ with the trained B, which strongly affects the output covariance (here for blue input patterns). This illustrates the importance of tuning the recurrent connectivity in shaping Q^1, as well as with the discrimination capability for Q^0.

Learning input spatio-temporal covariances

Now we consider the "converse" configuration of Fig. I-A where each input pattern is formed by a pair of non-zero P^0 and P^1, see Fig. II-A. The output is trained only using Q^0, meaning that the input spatio-temporal structure is mapped to an output spatial structure. This time simplifying Eq. (S1), the weight updates are given by Eq. (15) in the main text, which corresponds to discrete Lyapunov equations that can be solved at each optimization step to evaluate the weight update for A and B.

We first examine the specialization in terms of covariances in Q^0 as defined by the objectives in Fig. II-C. Here we take input patterns P^0 that are all identical (left matrices in Fig. II-B) such that the weight specialization must be based on the discrepancies between P^1 across inputs, even though this configuration may not be realistic for simulated time series. The desired outcome after training is obtained as illustrated in Fig. II-C. The surrogates (in black) indicate the importance of the trained recurrent connectivity A, although it appears less strong here than in Fig. I-F. Despite incidental troughs, the classification accuracy increases and eventually stabilizes around 90%. Second, Fig. II-D uses the same procedure for specializing the variances in Q^0 and shows similar conclusions. Together, these results demonstrate a useful flexibility in tuning the input-output covariance mapping using the MAR network.

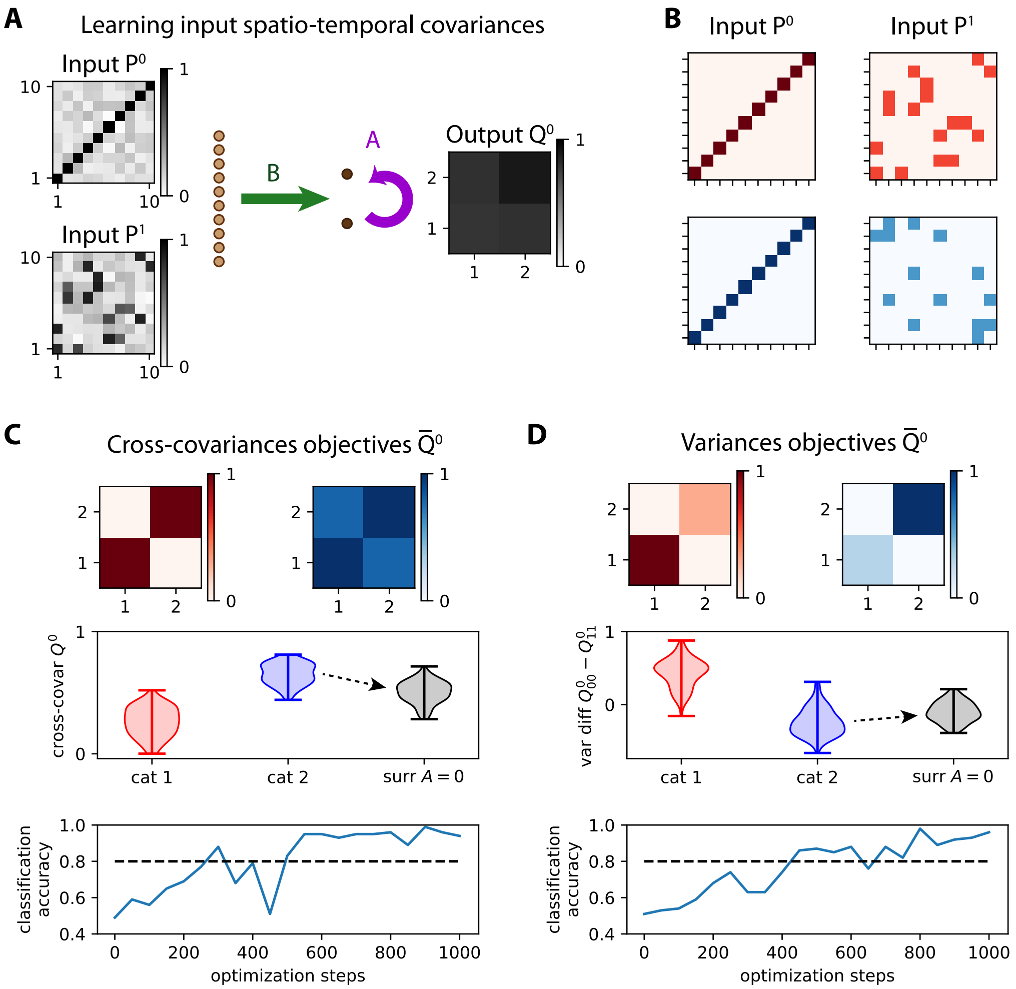

Fig II. Learning input spatio-temporal covariances with both afferent and recurrent connectivities. A: Similar network to Fig. I-A with $m = 10$ input nodes and $n = 2$ output nodes. **B:** Two examples of input patterns corresponding to a pair P^0 and P^1, among the 5 in each category. The P^0 matrices are identical for all patterns. **C:** Classification based on specializing cross-covariances for the two categories: absent for red and positive for blue (top matrices, same as Fig. 4D in the main text). The middle plot is similar to Fig. 4 (main text), where the separability of the red and blue distributions indicates the performance of the classification. The comparison between the black and blue distribution shows the importance of the recurrent connectivity A, which is forced to 0 for the surrogates. The bottom plot indicates the evolution of the classification accuracy during the optimization. The binary classification uses the same boundary as in Fig. 4E (main text). **D:** Same as panel C for specializing the variances of the output nodes, with the same objective matrices and classification procedure as in Fig. 4A-B (main text).
