# Supplementary material for: The covariance perceptron: A new paradigm for classification and processing of time series in recurrent neuronal networks
Source: PLoS Comput Biol. 2020 Oct 12;16(10):e1008127. doi: 10.1371/journal.pcbi.1008127 (PMC7595646; doi:10.1371/journal.pcbi.1008127)
Supplement: Attachment — Submitted filename: reply1.pdf [file pcbi.1008127.s002.pdf]

Matthieu Gilson
Universitat Pompeu Fabra, Barcelona, Spain
Institute of Neuroscience and Medicine (INM-6), Jülich Research Centre, Germany

Barcelona/Jülich, May 2020

Dear Dr Richards,

Please find attached the revision of our manuscript "The covariance perceptron: A new paradigm for classification and processing of time series in recurrent neuronal networks". We are very grateful for the insightful comments received by the three reviewers, as well as your highlighting the need to address two key points regarding the comparison with machine learning techniques and the motivation from the neuroscience viewpoint. We are convinced that the revised manuscript addresses all issues raised by the reviewers. Please find a point-to-point reply attached to this letter. For convenience, we colored changes of the manuscript.

With kind regards on behalf of all co-authors,

Matthieu Gilson

Reply to comments of the editor

(1) *It is especially important that you address the following issues raised by the reviewers:*

1) Reviewers 1 & 2 note some instances where the comparison to previous ML approaches is a bit impoverished. How do ML models with similar numbers of parameters perform? What do you gain beyond that which is gained by approaches that apply standard ML techniques to second-order statistics from the data? Is it really true that LSTMs or other approaches for time-series analysis cannot achieve what this model achieves?

We thank the editor for this concise summary of the reviewers' comments.

As requested by the reviewers, we ran new simulations to further compare our results with the logistic regression (MLR), enforcing the same number of resources (weights to optimize) for both classifiers that discriminate the moving MNIST digits. Limiting the resources causes the MLR performance to drop to around 81%. We also compared the performance with backpropagation through time applied to a recurrent neural network (RNN+BPTT), finding a slightly weaker performance for the RNN when the numbers of parameters is matched. In parallel, we relaxed some conditions on the objective to be learned (new Fig. 11B, tuning only the output variances that are used for classification of moving MNIST digits), which improved the performance by more than 10%, namely 71% compared to 59% before. Taken together, these results, summarized in the new Fig. 11C, show a difference in performance of less than 10% for the covariance perceptron, but still below the limited-resource MLR (presumably due to its nonlinearity) and the RNN.

The main objective of this work is to demonstrate a novel means of information processing based on the second statistical order that does not rely on the presence of a mean trajectory representing each class, which has neurobiological underpinnings. We have striven to compare the performance of this new scheme in a similar ballpark as established approaches of machine learning, but we cannot be exhaustive in a single paper. Therefore, we want to leave further comparison for future work, including the general question of finding the best regularities in such input time series for classification, like covariances versus temporal profiles.

(2) *2) Reviewer 3 was concerned about the motivation/contribution. If, as all the reviewers suspect, many of the things that this model can achieve can be achieved by existing ML techniques, then the motivation has to be a neuroscience one. But, as Reviewer 3 noted, the current neuroscience motivation is limited to some degree. Noise in the brain is potentially easily dealt with thanks to the large numbers of neurons in the brain, and STDP can be reconciled with means, as the reviewer described. Moreover, there is the issue of the biological plausibility of the weight updates that the reviewer highlighted. Arguably, the manuscript needs to do a better job of either (1) demonstrating some clear advantages over other existing ML techniques, or (2) motivating why second-order representations are an important possibility for computational neuroscientists to consider. Note: even if the learning rule is not currently biologically plausible, that's okay, but the motivation for why this should still be considered despite such a limitation has to be much stronger.*

Our study sits at the intersection of neuroscience and machine learning, but has a stronger neuroscience orientation as can be seen from the first three paragraphs of the Introduction. The connection between these two fields seems to be particularly active in PLoS Comput Biol with more than 400 articles since the start of 2019.

We formally (but respectfully) disagree with the statement by reviewer 3 that STDP can entirely be explained in terms of the first order statistics. In fact, the early work from Kempter, Gerstner and van Hemmen (Phys Rev E 1999) has shown that the weight structure in feed forward networks specializes according to spike covariances, not means of spiking rates (see their eqs. (2) and (3)). That theory was extended to recurrent networks by Gilson et al. (Biol Cybern 2009-2010). What matters is the collective behavior of input neurons that strongly affect STDP and the emerging weight structure (Gilson et al., Front Comput Neurosci 2010; Gilson et al. PLoS Comput Biol 2011). The paper by Ocker et al., mentioned by reviewer 3, follows the same line of thoughts. Our study can be seen as proposing a supervised counterpart for STDP. The fast-varying co-fluctuations of firing statistics used with STDP (Gilson et al. PLoS Comput Biol 2011) are of similar structure as the correlations found in the time series used in the present manuscript (we stress this in the revised text after Eqs 2-3). An important novel contribution of our work is to consistently formulate the problem of supervised learning with input and output covariances in a recurrent network, as was acknowledged by reviewers 1 ("I really like this idea of the self-consistent formulation and agree that it is essential for neuroscience to work towards this goal") and 2 ("The framework is quite general, and therefore it may have broad implications and utility").

About the plausibility of our proposed learning rule for recurrent connections, we are well aware that solving the Lyapunov equation does not give a local rule, as we said in our Discussion. We made new simulations (new Fig. 8, see also new Fig. 9G) to demonstrate that an approximation of the weight update that does not require solving the Lyapunov equation performs as well as the full gradient descent, proposed in the original manuscript. We also made a first step toward a biologically plausible learning rule by considering its calculation using only "information" available from neighboring neurons in the recurrent connectivity. Although the performance degrades compared to the full gradient descent, this approximation still works. This addresses the main point raised by reviewer 3 in our opinion.

Reply to comments of reviewer #1

(1) *I'd like to start my review by saying that this paper addresses some important and interesting problems. Namely, how to go beyond the typical neuroscientist and machine learning analysis of the means of time varying stimuli. Specifically, the approach here could be described as going from first to second order. It still uses an abstraction (means + covariances), but this includes at least some of the temporal information in the stimuli. This paper could be seen as a first step on the road to understanding how to use temporal information. In particular, the most interesting part of the paper for me was the suggestion in the discussion:*

"In contrast to the application of a measure to time series as a preprocessing step for machine-learning algorithms like the perceptron, our scheme opens the door to a self-consistent formulation of information processing of time series in recurrent networks, where the source signal and the classifier output have the same structure."

I really like this idea of the self-consistent formulation and agree that it is essential for neuroscience to work towards this goal.

With that said, the results and analysis presented here do feel like a very preliminary first step on this road only. Maybe that's necessary.

We thank the reviewer for sharing our enthusiasm in this topic. We agree that this is a first step, but an essential and fundamental one which demonstrates practical relevance, using both synthetic and "real" data (MNIST).

In addition, we have already expanded on the capacity of the covariance perceptron in a recently published paper in J Phys A (https://doi.org/10.1088/1751-8121/ab82dd), which was planned as a side-by-side publication. This publication shows the theoretical capabilities of the covariance perceptron in terms of performance, independent of the actual training. The current manuscript shows one way to practically implement this scheme, using a gradient-based learning rule.

Future work will be required to make the neuron and learning model more realistic (in particular, local learning rule, spiking neurons). We still feel that the conceptual developments presented in this first manuscript are required to allow the community to participate in this interesting endeavor.

(2) *Some comments in no particular order:*

One of the key claims made is about information compression and the fact that compared to using covariances as a preprocessing step in a more standard ML approach, they use many fewer parameters (nm+n^2 or O(m) where m is the number of inputs and n the number of outputs, as compared to O(m^2) if using the matrix of covariances as a preprocessing stage). This is stated explicitly at the top of page 21. This would be a very powerful point if the results were comparable. However, the ML method with more parameters has much higher performance (98% compared to 59%). It would be interesting and fairly quick to check if the results of the proposed method are better if the ML method is restricted to a similar number of parameters, for example by randomly selecting k entries in the covariance matrix with k selected so that the total number of parameters was about the same, and using MLR on only those k randomly selected entries. Would the MLR perform better or worse in this scenario?

We thank the reviewer for this suggestion that indeed provides an interesting comparison between the covariance perceptron and the MLR. As suggested, this new comparison is included in the new Fig 11C of the revised manuscript. After subsampling the input covariances to have the same number of weights to tune for the MLR as with the covariance perceptron, the MLR performance decreases:

"To match the number of resources, we repeat the MLR classification by randomly subsampling 180 distinct matrix elements of P^0, reducing the performance to 81%. The difference in performance of 10% between the covariance perceptron and the MLR with matched resources indicates that the covariance perceptron makes a efficient use of resources for the classification."

In addition, we have also improved the performance of the covariance perceptron, by only tuning the output variances used for classification (instead of the full output covariance matrix before). This increases the performance to 71%, still below, but closer to, the performance of the MLR with matched resources. These new insights are summarized in the text below that was added to the last Results section of the manuscript:

"A technical point here is discarding output cross-covariances in the training to only tune the output variances that are used for the classification. This improves the classification performance by more than 10% in the example of Fig. 11B (gray versus green traces). The result can be intuitively understood by the fact that cross-covariances add further constraints on the weights. In particular, enforcing zero cross-covariances between the output means decorrelating them "spatially", which is not primarily useful for classification."

(3) *I'd also be interested to know if the model could pick up on even higher order statistics. For example, if you designed input time series with equal means and covariances, but different higher order statistics, could it classify them or not? If so, that's a clear advantage over doing cross-correlation followed by standard ML stuff which by construction couldn't do this task. If not, then the strength of the method (purely in terms of performance) rests mainly on whether it makes more efficient use of the parameters it has.*

There is no particular reason to stop at any order in the statistics. In fact, a strength of our formalism is that it can be extended to higher orders. In addition, classification that is jointly based on both, means and covariances (or even higher order statistics), is a very interesting direction as mentioned in the Discussion. We would like to leave this direction for future work. We updated our discussion according to this highly interesting remark of the reviewer.

"The generalization to higher statistical orders seems a natural extension for the proposed mathematical formalism, but requires dedicated input structures and is thus left for future work."

(4) *It seems MNIST was used in the following way: the two spatial dimensions of the digits were converted into one spatial and one temporal dimension. This feels like an odd way to construct time-varying stimuli. Why not use a more natural time-varying stimuli like an auditory signal?*

We apologize for not making clear enough that we are using time-varying stimuli in this task by choosing moving digits rather than static MNIST. In the considered setup, a two-dimensional receptor array was swept over the digits. Technically, this process converts the temporal information (movement direction of the digit) into spatial covariances between receptors in different columns. This example of moving digits was motivated by making a modest step in the direction of computer vision for video, for which neural networks (even deep) still do not exhibit the excellent performances that they do for images . We thus designed this example with moving digits as a multimodal detection task where information embedded in spatial covariances about both digits and motion should be used for classification.

The following sentence has been added to avoid confusions:

"The image of the digit is swept over a two-dimensional receptor array. As a result, information about both motion direction and digit identity is transformed into only spatial covariances between the inputs."

and the following sentences have been rewritten for better clarity about our motivation:

"The goal of this demonstration is not so much to find the best classification tool, but to see how our proposed learning and classification scheme performs with "real-life" data, in particular when the Gaussian assumption for inputs is violated."and

"... both variances and cross-covariances are required for correct classification, the former being strongly related to the digit identity and the latter to the motion direction. We now test whether the covariance perceptron can efficiently extract the relevant information from the second-order statistics of those patterns, while comparing it to other classification networks."

(5) *p16: "To our knowledge this is the first study that tunes the recurrent connectivity in a supervised manner to specifically extract temporal information when spatial information is "absent"." Really? I'd guess that LSTMs (for example) can distinguish between different temporal patterns with the same means. Maybe I'm misunderstanding something here?*

This is a misunderstanding due to the specific meaning that we put in "temporal and spatial information". We agree that the sentence without this context should be amended for better clarity. As exposed at the beginning of Section 4.2, "information" refers to covariances in that sentence. Particularly we use "spatial information" to denote the zero-lag covariances and "temporal information" for time-lagged covariances. We have thus reformulated the sentence as:

"To our knowledge this is the first study that tunes recurrent connectivity in a supervised manner to specifically extract temporal information from lagged covariances when spatial covariances are not informative about the input categories."

(6) *p21: "It is left for future work to test whether the mean trajectories as those in Fig. 9C can be used for prediction, using classifiers for time series like long-short-term memory units." Why not just do it? I would guess this wouldn't take that long to check with a fairly standard ML toolkit?*

We have added in the new Fig 11C a comparison of the performance of a recurrent neural network (RNN) trained with "vanilla" back-propagation through time (BPTT). The two versions were chosen to match either the number of resources or the performance (see also Fig 11E). The conclusion of this comparison is that the covariance perceptron achieves a better classification performance with a small number of resources.

(7) *p22: "Because our training scheme is based on the same input properties, we expect that the strengths exhibited by those learning rules also partly apply to our setting, for example the robustness for the detection of time-warped patterns as studied in [5]." I think this statement needs some more evidence to back it up or should be toned down.*

We have amended the sentence to highlight what we think is an interesting direction, without strong claim on the potential outcome:

"Because the classification of time-warped patterns can be based on the second-order statistics of the input signals (**?**), we foresee a potential application of our supervised learning scheme, as the time-warping transformation preserves the relative structure of covariances between the input signals (albeit not their absolute values)."

(8) *I don't know the editorial policy on providing data and code for modelling studies like this. I think it would be great if the authors made their code available, as I suspect it would be rather difficult to fully reproduce their analyses from the text alone (which seems to leave some details out).*

This is a good point and we had the intention of making the code publicly available, as we did with previous studies (https://github.com/MatthieuGilson). For the current manuscript, we are planning to release the basic code to replicate the important figures, then later we will wrap up the code in a clean package.

(9) *Final thing to note is that I've suggested some additional work to do in my review above. Since we are currently in the middle of a global crisis that is severely impacting on everyone's time and ability to work, I would like these to be taken only as suggestions if the authors have time and capability to do them at the moment, but that is a choice for the editors to make.*

We thank the reviewer again for taking the time to assess our manuscript, especially during these days, where business as usual is hardly possible. We are very thankful for the very deep and insightful remarks by the reviewer and we are certain that these helped in considerably improving our manuscript.

Reply to comments of reviewer #2

(1) *The authors introduce a new theoretical framework for training linear recurrent neural networks to map multivariate input covariances into output covariances. They show that their framework can learn to classify input covariance patterns, by mapping input covariances to output nodes, whose covariance is used to identify input patterns. They extend their results to learning lag-1 temporal dynamics. They also explore the finite-sample characteristics of their approach, and the network capacity.*

General comments:

This manuscript presents an interesting extension of ideas developed for perceptrons, to second order statistics. The framework is quite general, and therefore it may have broad implications and utility. I found the presentation to be relatively clear and straightforward, and the authors did a good job drawing out interesting applications of their approach.

We thank the reviewer for the concise summary and the helpful critique provided below. We are glad that the reviewer shares our interest in this topic and appreciates the presentation of our results.

(2) *They link their work to some other previous work, including using logistic regression on covariances. Perhaps a bit more development in this direction would be useful.*

Further simulations are now presented to compare the performance of the MLR with the same number of resources (e.g. weights to train) as the covariance perceptron. We achieve identical numbers of parameters in the MLR by randomly selecting input covariance matrix elements. The result is that the performance drops from almost 100% to around 81% (see new Fig. 11C). Meanwhile, the training of the covariance perceptron with relaxed conditions on the objective (namely using only the variances on the diagonal of Q^0, which are used for the classification, while letting the cross-covariances unconstrained) gives a classification performance around 72%. In that sense, the use of the resources by the two methods are comparable, and the superior performance of the MLR is believed to come from its nonlinearity, while our conceptual presentation here focuses on a linear mapping only. We also compared the covariance perceptron with a recurrent neural network (RNN) trained using backpropagation through time (BPTT), which yields a classification performance that is close, but below that of the covariance perceptron for the same number of resources ($h = 6$). Together, these further simulations lead to the conclusion that the covariance perceptron, in its current state as a proof of concept, performs reasonably well compared to these established machine learning techniques.

(3) *Specific comments:*

1. Although the authors develop their framework in the general context of mapping from input covariances to output covariances, some mappings would not be possible. If I understand correctly, for example, if P0 lacks the modes that one would like to see in Q0, I do not believe a B could be found that would realize the mapping. Some brief discussion of this would be useful.

We agree that a mapping $Q^0 = BP^0B^\mathrm{T}$ is not always achievable, even for a single input pattern. For example, if the rank of P^0 is smaller than the rank of Q^0, this equation has not strict solution. If the rank of P^0 is larger than or equal to the rank of Q^0, such a mapping can always be found. This an be seen by diagonalizing $U^\mathrm{T}P^0U = \Lambda$ and $V^\mathrm{T}Q^0V = M$, where Λ and M are diagonal with non-negative entries. The matrix B can then be constructed as

$$B = \sum_{1\leq i\leq \mathrm{rank}(\mathrm{Q}^0)} V_i \sqrt{\frac{M_i}{\Lambda_i}}\, U_i^\mathrm{T},$$

where we assumed the non-zero eigenvalues to be the first $\mathrm{rank}(Q^0)$ ones.

In more general cases solution do not exist, as was shown in Fig. 7, the gradient descent aims to reduce the error and the weights stabilize in the best possible manifold for that purpose. We have added the following sentence in the Discussion:

"As with the classical perceptron, classification relies on shaping the input-output mapping, for example by potentiating afferent weights from an input with high variance to two outputs to generate correlated activity between the outputs. Note that, in general, the existence of an achievable mapping between the input patterns and the objective patterns is not guaranteed, even when tuning only afferent connections with $\bar{Q}^0$. Nonetheless, the weight optimization aims to find the best solution as measured by the matrix distance with respect to the objectives (Fig. 7)."

(4) *2. The approach in the manuscript is developed in the context of recognizing covariance structure in the input. A fair bit of work has been done in this area, in speech recognition systems. Although these systems generally use a pre-processing step that computes some sort of Fourier or other transform on the inputs. Old approaches to this problem tended to follow the Fourier transform step with a HMM step that computed the probability of observing a given time series, under each of many HMMs that recognized different patterns, for example phonemes. I wonder if the current results can be related to some of these other approaches for doing classification on second order statistics? What are the pros and cons? Perhaps there are broader uses of the current results, beyond classification, that can be considered? Also, instead of using a pre-processing step that effectively computed the covariances, some approaches suggested learning Kalman filters, one for each category to be recognized, and then computing the probability of observing individual time-series from each Kalman filter. How would the current approach relate to those approaches?*

We thank the reviewer for pointing out this interesting direction for future applications and for testing our formalism. In our understanding, a processing pipeline such as that described by the reviewer involves two parts: dynamic feature detector for phonemes (implemented by HMM in the reviewer's example) and characterization of phoneme streams that form words (then several words forming sentences; here tracking algorithms like the Kalman filter could presumably detect sequences whose phoneme structure are encoded in the filter dynamics). There are thus two timescales here, phonemes and sequences thereof. In this picture, the first application for our formalism would rather be to the first part, by detecting phonemes, whose spectral structures (akin to covariances) are characteristic of phonemes. The applicability to sequences at the slower timescale entirely relies on whether phoneme sequences could be described by covariances evaluated at that timescale (presumably with time lags to capture transitions). We think that such considerations are an interesting perspective, but still too speculative to be incorporated to our manuscript.

Reply to comments of reviewer #3

(1) *In this paper, the authors propose what I believe is a fundamentally new approach to learning: adjust weights to achieve a target lagged covariance matrix among neurons in a population. They derive learning rules, and show that the scheme can perform reasonably well when classifying moving MNIST digits.*

We thank the reviewer for highlighting the novelty of our approach.

(2) *The work seems solid, and the idea is interesting, but it's not clear to me that it's relevant for either neuroscience or machine learning. The motivation seems to be that neurons are noisy, so classification based on means may not be a good idea. That's what I extracted from the second and third paragraphs of the introduction, but I will admit that I did not fully agree with the logic. For instance, the first sentence of the second paragraph of the introduction reads*

"The representation of the stimulus identity by the mean firing activity alone is, however, challenged by two observations in biological neuronal networks."

The two observations were that synaptic plasticity depends on pre-post timing and activity in cortex is variable. But the latter doesn't argue very strongly against mean activity being a bad representation; in this day and age, noise is not hard to deal with. Spike timing dependent plasticity is somewhat more problematic for mean-based rules, although when learning rates are low it can be cast as a firing rate covariance rule (see Ocker et al., PLOS CB 11:e1004458, 2015). So it also doesn't provide a strong argument that the mean is a bad representation.

We think that this interpretation of our approach by the reviewer is a misunderstanding: our intention was not to say that mean-based classification is "bad". We instead wanted to highlight the difference of our approach with respect to mean-based (de)coding, presenting our scheme more as an alternative.

From the Ocker et al.'s paper (PLoS Comput Biol 2015), we do not deduce that STDP is sensitive only to the mean. Ocker et al.'s Discussion starts with "The resulting system naturally classifies STDP rules into two categories: 1) rules with an imbalance between potentiation and depression and plasticity dominated by the firing rates of neurons in the network, and 2) rules with balanced potentiation and depression in which different sources of spiking covariance interact with the STDP rule to determine network structure. In the latter case, the importance of spiking covariances due to forwards connections, reciprocal connections, and common inputs creates new equilibrium points for the weighted motif structure of the network." This only means that some STDP parametrizations are dominated by firing rates, but not that any STDP rule can be reconciled with a rate formulation. On the contrary, Ocker et al. pursue their discussion with effect of spike covariances on the weight structure, which has been the focus on many STDP studies.

The fact mentioned by the reviewer that Ocker et al. describe the learning as a "firing rate covariance rule" is completely in line with the view presented here: The multivariate Gaussian process that we study has in fact been shown to have the same covariance structure as networks of spiking neurons, approximated in linear response (Grytskyy et al. 2013).

We have amended the Introduction to make this point clearer:

"Examples of second-order statistics of the spiking activity that induce strong weight specialization not only include the canonical example of spike patterns with reliable latencies, like spike volleys following visual stimulation (Masquelier et al., PLoS One 2008), but also a great variety of spiking statistics such as fast stereotypical co-fluctuations even in the case of Poisson-like firing (Gilson et al., PLoS Comput Biol 2011). Nonetheless, the common feature to all those input structures is the collective spiking behavior."

(3) *Motivation aside, one might ask about biological plausibility. As the authors point out in the discussion, this is an open question. But it seems worse than that: Methods, Eq. 26, indicates that to compute the weight updates it's necessary to solve a discrete Lyapunov equation on every timestep. It's hard to imagine how neurons could do that. In fact, the complexity scales as n^3, so for large populations even computers have problems.*

We agree that the proposed learning rule in the submitted manuscript was not biologically plausible nor optimized for performance. Our point was to demonstrate a proof of principle that the covariance perceptron can be practically relevant for synthetic data and "real-life" data (MNIST digits). Following the reviewer's comments, we have explored in the revised manuscript two approximations for our covariance-based learning rule (new Section 4.2 in the revised manuscript). The first is a computational approximation for the solution of the Lyapunov equation, which avoids the N^3-complexity of computation and simply relies on matrix multiplications. We show that this approximation for the simultaneous training of afferent and recurrent connections performs as well as the full gradient descent that involves solving the Lyapunov equation, as illustrated in Figs. 8 and 9F of the revised manuscript (time series classification).

The second (and additional) approximation examined in Fig. 8 is a step toward biological plausibility. The approximation only considers quantities that are accessible from the output of connected neurons for the calculation of the weight updates, namely via presynaptic connections in the A matrix. Of course, this only makes a difference when the connectivity matrix A is "sparse" (i.e. not full) and Fig 8 shows that the approximation performs reasonably well. We leave for future work further refinement of this approximation to make it fully online and biologically plausible.

Finally, we would like to mention the results in the recently published sister paper in Journal of Physics A that examines the capacity of the covariance perceptron, namely how many patterns can be discriminated. That study shows that, per synapse to a single readout, 4 patterns in general position can be discriminated, to be compared with 2 such patterns for the classical perceptron.

(4) *The authors do show that their method can do an OK job classifying moving digits, and it does better than the mean-based models they looked at. However, I would be shocked if an RNN wasn't perfect on this task. So it's not an obvious advance over standard networks.*

Our motivation is not to compete with pure ML techniques here, but to get closer to biology. This is the reason behind our choice of inputs to discriminate, which involve large (and structured) variability. For the MNIST dataset, the input activity trajectories can also be used by the RNN with backpropagation through time (BPTT) to perform classification with good performance (see new Fig. 11 C).

However, the information extracted by the covariance is different in nature compared to the RNN+BPTT: While the RNN can (and does) rely on the temporal sequence of the mean stimulus, the covariance perceptron performs the classification purely based on the equal-time second order statistics. We would like to leave a more thorough comparison between the covariance perceptron and BPTT to future work, as the present manuscript is already quite long. Still, provided that this manuscript presents a new concept for supervised learning based on second order statistics, we feel that a proof of principle for its operation is of course a necessary but also sufficient criterion to be of potential interest. The question whether or not this paradigm outperforms ML techniques given the same amount of resources is interesting from a technical point of view. But given the motivation to find paradigms that are potentially employed in the brain, the latter question to us seems of subordinate importance. More so, since other factors, such as fault tolerance, robustness to noise and biological variability may substantially bias a comparison between paradigms beyond the mere comparison of resources alone. Lastly, having done the conceptual work in this manuscript, it is quite likely that subsequent work may find improvements that in fact may render its performance higher on a set of given tasks than traditional approaches.

(5) *Overall, then, I would say it's an interesting idea, but there do not seem to be any potential applications to either neuroscience or machine learning.*

Here we respectfully disagree with the reviewer's conclusion. As we argued above (and in the Discussion), our approach introduces a novel concept for supervised learning in recurrent neuronal networks, based on the transformation of the second-order statistical properties of time series, that is consistent with regard to the input-output mapping. We strongly believe that this type of processing will inspire interesting developments, both in neuroscience and machine learning. This assessment is shared by reviewer 1 ("This paper could be seen as a first step on the road to understanding how to use temporal information.") and by reviewer 2 ("The framework is quite general, and therefore it may have broad implications and utility.").

(6) *Minor comment: it wasn't clear to me what rule was used on the moving digits problem. But may be I missed it?*

The training of afferent connections for the MNIST dataset (Figs 10 and 11 in the revised manuscript) is similar to that in the Fig 3 and relies on Eq 10. We have added this description in the caption of Fig 10 and in the corresponding text.

Finally, we thank the reviewer for the critical assessment of our manuscript and the very helpful ideas of comparing it to existing approaches. As a result, in particular having demonstrated the biological implementability of the approximate learning rule, we feel that the manuscript has been strengthened considerably compared to the initial submission.
