# Supplementary material for: The covariance perceptron: A new paradigm for classification and processing of time series in recurrent neuronal networks
Source: PLoS Comput Biol. 2020 Oct 12;16(10):e1008127. doi: 10.1371/journal.pcbi.1008127 (PMC7595646; doi:10.1371/journal.pcbi.1008127)
Supplement: Attachment — Submitted filename: response_R2.pdf [file pcbi.1008127.s003.pdf]

We thank again the reviewers for their comments and appreciation of our revised manuscript.

Reviewer #1: My thanks to the authors for their careful response to my review.

This revision addresses all my technical points about this manuscript, although I do think that the way the part about the number of parameters compared to MLR is written (p23) is still a bit misleading. I think it would be better to come clean that it doesn't do as well as MLR even with the same number of parameters, but may have other advantages.

Even though the case made for the covariance perceptron is not overwhelming in my opinion (since it isn't obviously an improvement in terms of machine learning, and biological plausibility is unclear), I'm supportive of publishing this paper, and more of this sort of work that tries to go beyond standard rate-based learning methods.

We thank the reviewer for the appreciation of our work and its conceptual originality. The text p 23 has been revised according to the above comment:
"The performance of the covariance perceptron is thus about 10% lower than that of the MLR with matched resources, which presumably comes from the non-linearity of the MLR. To further check the importance of the non-linearity, we repeat the experiment with a linear regressor instead of the MLR and the performance becomes 84% using the full covariance matrix and 1530 weights to train, but drops to 48% for matched resources with 180 weights. This indicates that the covariance perceptron makes efficient use of resources for the classification based on a linear mapping between the input and output covariances."

Reviewer #3: I don't feel massively strongly about this paper, but I am still not convinced about the locality of the learning rules. Take, for instance, the local learning rule in Fig. 8. Assuming I understand things, the learning rule is (when P^{-1}=0) something like

Delta A_ij \propto sum_kl delta Q_ik A_kl .

(It would be really nice if this were written down somewhere, since it's an important equation.) If we make the rather extreme approximation that

sum_l A_kl y_l^t = y_k^(t+1},

we have

Delta A_ij \propto sum_k delta Q_ik .

The term in brackets could be computed with a running average, but that term is stored in synapse j on neuron k. Even if the sum over k is restricted to connected pairs, I still don't see how that information can be transferred to synapse j on neuron i. Without at least some sort of plan for doing that, this just does not seem relevant for neuroscience. And to make things worse, that's a very extreme approximation, so even if the above update rule could be computed locally, it's not clear that performance would be good.

If this paper is published, the authors should be _very_ clear about exactly how local the learning rule is.

We have incorporated the recommendation of the reviewer in the new Eq (17) that rewrites the learning rule for recurrent connections. The expression in Eq (17) shows that "the necessary

information for computing the weight update is the summed activity received by the parents neurons i', which has to be centralized by the downstream i''". This approximated rule is "local in the graph in the sense that it only uses information from neighbor (parent) neurons".

Also, three minor comments.

1. The authors seem to imply that noise is a problem for rate-based approaches, but not for the approach advocated here. However, the covariance has to be estimated from data, and second order statistics are, I believe, more sensitive to noise than first order statistics. So this approach does not seem to have an advantage with respect to noise. But maybe I misinterpreted their claim?

The misunderstanding seems to come from the fact that, in the model, the covariances are the basis of information that is processed, not that "the covariance has to be estimated from data" as in an inference context. The point of Fig 5 is to show that, even though covariances may be noisy to estimate, they can be processed to robustly convey relevant information (here learn to classify input patterns).
About the pointed passage in Introduction about noise and rate-based representations, we have added the following first sentence about the large variability observed in experiments and reorganized the text to highlight why information may be based on this variability instead of spiking rate:
"Rate-based representations and learning rules address the issue of noisy inputs by averaging activity over time or across neurons, thus considering this variability as noise. Previous studies have proposed that this noise may have a functional role related to probabilistic representations of the environment in a Bayesian framework [Berkes Science 2011, Orban Neuron 2016]. However, the spiking variability has also been closely linked to behavior [Riehle Science 1997, Kilavik J Neurosci 2009, Shahidi Nat Neurosci 2019]. Experimental and theoretical evidence thus points to a relation between the variability of neuronal activity and the representation of the stimulus."
Our proposal is a different representation of stimuli than "by the mean firing activity alone" (as we say at the beginning of the corresponding paragraph in Introduction), but this doesn't mean that rate-based representations are bad.

2. I didn't really understand Fig. 3. How many different patterns are being trained? All the covariance matrices look about the same to me.

In Fig 3C and D, each category corresponds to 5 covariance patterns that are displayed in the left column of each figure. The training is performed on noisy versions of those patterns, as represented in Fig 3A. The initial images of the patterns are very similar before training (middle column), but the variance become specialized by the training. The following sentence has been added to the caption:
"The training leads to a larger variance (darker pixel) for output 1 (bottom left pixel of matrices in the right column) than for output 2 (top right pixel)."

3. I found this paper really hard to read. Maybe it's fundamentally hard, but I don't think so. Unfortunately, I don't really understand things well enough to make concrete suggestions. Except to write down the learning rules as I did above; at least it's possible to look at them and figure out what has to be computed.

We have striven to be didactic about the type of information that can be embedded in time series (Figs 1 and 2). Then we present the basic concepts step-by-step about the tuning of the input-output mapping for covariances in feedforward networks (Fig 3) and how this can be used for classification (Figs 4 to 6), with the practical application in Figs 10 and 11. Figs 7 to 9 show how

the same scheme applies to recurrent networks and, among them, the study how to approximate the learning rule using a local rule in Fig 8 is a first step in that direction. Therefore the majority of the calculations concern matrix equations that show the consistency of the calculations in all cases from Figs 3 to 11. As explained above, we have also incorporated the recommendation of the reviewer in the new Eq (17) as a first step toward a local learning rule.
